# Exploring the potential of isorhapontigenin: attenuating *Staphylococcus aureus* virulence through MgrA-mediated regulation

**DOI:** 10.1128/msphere.00317-24

**Published:** 2024-06-05

**Authors:** Lei Yuan, Huimin Xi, Zhaoxia Luo, Mei-fang Liu, Qiang Chen, Qing Zhu, Rui Zhao, Yi-yun Sheng

**Affiliations:** 1 Department of Clinical Laboratory, Medical Center of Burn plastic and wound repair, The First Affiliated Hospital, Jiangxi Medical College, Nanchang University, Nanchang, China; 2 Department of Pulmonary and Critical Care Medicine, The First Affiliated Hospital, Jiangxi Medical College, Nanchang University, Nanchang, China; 3 Department of Pathology, The First Affiliated Hospital, Jiangxi Medical College, Nanchang University, Nanchang, China; University of Nebraska Medical Center College of Medicine, Omaha, Nebraska, USA

**Keywords:** *Staphylococcus aureus*, isorhapontigenin, MgrA, virulence

## Abstract

The emerging prevalence of drug-resistant *Staphylococcus aureus* isolates underscores the urgent need for alternative therapeutic strategies due to the declining effectiveness of traditional antibiotics in clinical settings. MgrA, a key virulence regulator in *S. aureus*, orchestrates the expression of numerous virulence factors. Here, we report the discovery of isorhapontigenin, a methoxylated analog of resveratrol, as a potential anti-virulence agent against *S. aureus*. Isorhapontigenin effectively inhibits the hemolytic activity of *S. aureus* in a non-bactericidal manner. Additionally, it significantly reduces the cytotoxicity of *S. aureus* and impairs its ability to survive in macrophages. Mechanistically, isorhapontigenin modulates the expression of virulence factors, dose-dependently downregulating *hla* and upregulating the MgrA-regulated gene *spa*. Electrophoretic mobility shift assays demonstrated that isorhapontigenin inhibits the binding of MgrA to the *hla* promoter in a dose-dependent manner. Thermal shift assays confirmed the direct interaction between isorhapontigenin and the MgrA protein. The *in vivo* experiments demonstrated that isorhapontigenin significantly reduced the area of skin abscesses and improved survival in a pneumonia model while decreasing bacterial burden and inflammation in the lungs. In conclusion, isorhapontigenin holds potential as a candidate drug for further development as an anti-virulence agent for treating *S. aureus* infections.

**IMPORTANCE:**

The emergence of antibiotic-resistant *Staphylococcus aureus* strains presents a formidable challenge to public health, necessitating novel approaches in combating these pathogens. Traditional antibiotics are becoming increasingly ineffective, leading to a pressing need for innovative therapeutic strategies. In this study, targeting virulence factors that play a crucial role in the pathogenesis of bacterial infections offers a promising alternative to circumvent resistance mechanisms. The discovery of isorhapontigenin as an inhibitor of *S. aureus* virulence represents a significant advance in anti-virulence therapy.

## INTRODUCTION

*Staphylococcus aureus* is a Gram-positive pathogen responsible for a wide array of infections in both humans and animals, ranging from mild skin conditions to life-threatening diseases, including endocarditis, pneumonia, and sepsis (1). In recent years, the emergence and widespread dissemination of methicillin-resistant *S. aureus* (MRSA) strains have posed a severe threat to public health (2). As the effectiveness of traditional antibiotics wanes in treating *S. aureus*-related infections, the exploration of novel drug targets has become an imperative strategy in the development of antimicrobial agents (3).

The infectivity and pathogenicity of *S. aureus* are dependent on a variety of virulence factors produced by the bacterium (1). These factors constitute an intricate regulatory network, adept at promptly responding to environmental signal changes (4). MgrA, a global transcriptional regulator in *S. aureus*, plays a significant role in the regulation of numerous virulence factors, co-regulating the expression of approximately 350 genes, including those encoding for α-hemolysin (Hla), adhesins, and proteases (5, 6). MgrA is pivotal in the *S. aureus* infection process, as evidenced by MgrA-deficient mutants demonstrating markedly reduced virulence in murine abscess models (7). Notably, MgrA is not essential for bacterial survival, suggesting that drugs targeting MgrA’s functions could effectively treat infections without exerting selective pressure that often drives the development of resistance (8, 9). Currently, there are limited reports on MgrA inhibitors, primarily focusing on small molecules and natural plant products (10).

Previous studies have demonstrated that resveratrol diminishes the expression of Hla, thereby attenuating the virulence of *S. aureus* (11, 12). Isorhapontigenin, a methoxylated derivative of resveratrol, can be extracted from commonly utilized herbal medicines (13). Compared to resveratrol, isorhapontigenin has demonstrated superior pharmacokinetic properties (14). In various models, isorhapontigenin has exhibited promising antioxidant and anticancer properties (15–17). Recent studies have revealed its inhibitory effects on several critical inflammatory signaling pathways, such as nuclear factor kappa B, activator protein-1, and PI3K/Akt (18, 19). Previous research indicates that isorhapontigenin does not possess significant antibacterial activity against *S. aureus*, with minimum inhibitory concentrations exceeding 128 µg/mL (495.6 µM) (20). However, the effect of isorhapontigenin on the virulence of *S. aureus* remains unexplored.

Given the protracted and costly process of developing new antimicrobial agents, drug repurposing offers an avenue to expedite research timelines and reduce costs by potentially circumventing extensive pharmacokinetic and toxicity studies (21, 22). The present study aims to evaluate the anti-virulence potential of isorhapontigenin against *S. aureus* and to investigate its effects on the major virulence factors. Additionally, this research seeks to ascertain the therapeutic efficacy of isorhapontigenin in treating *S. aureus*-induced skin abscesses and murine pneumonia models.

## MATERIALS AND METHODS

### Bacterial strains, cells, and culture conditions

The *S. aureus* Newman strain was preserved in our laboratory (23). Five methicillin-sensitive *S. aureus* (MSSA) strains and five MRSA strains, all exhibiting strong hemolytic activity, were randomly selected from clinical isolates at the Clinical Microbiology Laboratory of the First Affiliated Hospital of Nanchang University. Each isolate was identified utilizing the matrix-assisted laser desorption/ionization time-of-flight (MALDI-TOF) mass spectrometry system (Bruker Daltonics, Billerica, MA, USA). Antimicrobial susceptibility testing and interpretation were performed using the VITEK 2 automated system (bioMérieux, France) in accordance with the Clinical and Laboratory Standards Institute breakpoints (document M100-S32). *S. aureus* strains were cultured in tryptic soy broth (TSB) at 37°C with shaking at 220 rpm, except where noted otherwise. BEAS-2B (human bronchial epithelial cells) and L-02 cells (human liver cells) were purchased from American Type Culture Collection (Rockville, MD, USA). RAW264.7 cells, a murine macrophage-like cell line, were maintained in our laboratory. Unless otherwise specified, cells were cultured in Dulbecco’s modified Eagle medium (DMEM) supplemented with 10% fetal bovine serum and incubated at standard conditions of 37°C and 5% CO_2_.

### Chemicals

Isorhapontigenin (cat. no: HY-N2593, purity: ≥99.82%) was purchased from MedChemExpress (Shanghai, China). Dimethyl sulfoxide (DMSO) was used to dissolve isorhapontigenin (DMSO <0.1% in all experiments).

### Hemolytic activity of the isorhapontigenin

The hemolytic potential of isorhapontigenin was assessed using defibrinated rabbit red blood cells (RBCs) supplied by Nanjing Maojie Microbial Technology Co., Ltd. (Nanjing, China). Isorhapontigenin was diluted in phosphate-buffered saline (PBS). In separate tubes, a 100 µL aliquot of each isorhapontigenin dilution was mixed with 900 µL of PBS containing 5% RBCs to achieve final isorhapontigenin concentrations of 1, 25, 50, and 100 µM. The positive control comprised 2% Triton X-100, and the negative control was PBS. Following a 1-h incubation at 37°C, the mixtures were centrifuged at 4,000 × *g* for 5 minutes. The absorbance of the resulting supernatant was determined at 600 nm to calculate the hemolysis rate. This procedure was replicated three times to ensure reproducibility.

### Cell viability assays

Cell viability was evaluated using the 3-(4,5-dimethylthiazol-2-yl)-2,5-diphenyltetrazolium bromide (MTT) cell proliferation and cytotoxicity assay kit (E606334, Sangon Biotech, China). BEAS-2B and L-02 cells were plated at a density of 5 × 10^3^ cells per well in DMEM supplemented with 10% fetal bovine serum in the 96-well plates. The cells were exposed to varying concentrations of isorhapontigenin, or to 0.1% DMSO as a vehicle control. Following a 24-h incubation period, the wells were carefully rinsed twice with sterile PBS. Subsequently, 100 µL of fresh DMEM and 10 µL of MTT reagent at 5 mg/mL concentration were added to each well, taking care to minimize light exposure. Post a 4-h incubation at 37°C in a CO_₂_ incubator, the optical density at 600 nm was recorded using a microplate reader. This assay was conducted in triplicate.

### *In vivo* toxicity assay in *Galleria mellonella*

Larvae of *G. mellonella*, each weighing approximately 250 mg, were randomly allocated into five treatment groups (*n* = 10 per group), and each was administered with 10 µL of isorhapontigenin solution, prepared in sterile PBS, at doses of 5, 10, and 20 mg/kg body weight. A separate negative control group was inoculated with 10 µL of sterile PBS alone, and a positive control group received 10 µL of *S. aureus* Newman cultures (optical density at 600 nm [OD_600_] = 0.1). Post-injection, larvae were carefully placed into sterile Petri dishes and incubated in the dark at 37°C. Survival rates were assessed at 12-h intervals over a period of 5 days following the injection.

### Determination of minimum inhibitory concentration (MIC)

The MIC of isorhapontigenin against *S. aureus* strains was determined using the microbroth dilution method. Isorhapontigenin was serially diluted twofold in Mueller-Hinton (MH) broth within a 96-well microtiter plate, achieving concentrations ranging from 1 to 500 µM. Overnight cultures of *S. aureus* were diluted in MH broth to a final inoculum density of 1.5 × 10^6^ colony-forming units (CFU) per milliliter. This suspension was then added to the wells containing isorhapontigenin dilutions. Controls included a growth control without isorhapontigenin and a sterility control containing only MH broth. Following a 24-h incubation at 37°C, the MIC was identified as the lowest concentration of isorhapontigenin at which bacterial growth was visibly inhibited. The experiment was performed in triplicate to ensure reproducibility.

### Hemolytic activity assay

The hemolytic activity of *S. aureus* was assessed using defibrinated rabbit RBCs supplied by Nanjing Maojie Microbial Technology Co., Ltd. (Nanjing, China). *S. aureus* was cultured in TSB supplemented with varying concentrations of isorhapontigenin. Following a 24-h incubation period, the cultures were centrifuged at 8,000 rpm for 5 minutes at room temperature to obtain the supernatant. Subsequently, 100 µL of the supernatant was mixed with 900 µL of a 5% rabbit RBC suspension in PBS. The positive control containing 2% Triton X-100 was included, and the negative control contained PBS. After incubating the mixtures at 37°C for 1 h, they were centrifuged at 4,000 *g* for 5 minutes to separate the RBCs from the supernatant. The absorbance of the supernatant was measured at 600 nm, and hemolysis rates were calculated.

### Growth curves

*S. aureus* Newman culture was grown to the logarithmic phase and diluted 1:200 in TSB. The diluted cultures were incubated at 37°C with shaking at 220 rpm in the presence of isorhapontigenin at concentrations of 0 (control) and 50 µM. A sterility control containing only TSB was included. Bacterial growth was monitored by measuring the OD_600_. The experiment was performed in triplicate to ensure reproducibility.

### Western blot

*S. aureus* cultures with an initial optical density at OD_600_ of 0.3 were treated with isorhapontigenin at concentrations of 0 (control), 1, 25, and 50 µM and grown until reaching 24 h. The supernatants were collected, and total protein concentrations were quantified using the Bradford assay to ensure equal loading of samples on sodium dodecyl sulfate-polyacrylamide gel electrophoresis (SDS-PAGE). Protein samples were mixed with Protein Sample Loading Buffer (P0015, Biyuntian, China) and heated at 95°C for 10 minutes. After separation by 12.5% SDS-PAGE, the proteins were transferred onto a polyvinylidene difluoride membrane. The membrane was blocked overnight at 4°C with 5% skim milk and then incubated with rabbit anti-*hla* IgG antibody (Sigma) diluted 1:3,000. Subsequently, the membrane was incubated with goat anti-rabbit IgG horseradish peroxidase secondary antibody (Biosharp) diluted 1:5,000. Chemiluminescent detection was performed using the BeyoECL Plus Chemiluminescence Kit (P0018S, Beyotime, China).

### Infection of BEAS-2B cells and lactate dehydrogenase (LDH) assay

BEAS-2B human lung epithelial cells were seeded in 24-well plates at a density of 1 × 10^5^ cells per well and incubated in DMEM medium supplemented with 10% fetal bovine serum at 37°C and 5% CO_2_ for 24 h. Upon reaching 80% confluency, the culture medium was replaced with fresh DMEM containing different concentrations of isorhapontigenin (0, 1, 25, and 50 µM) and 50 µL of *S. aureus* suspension (OD_600_ = 0.5) to a final volume of 150 µL per well. The plates were incubated at 37°C for an additional 6 h. Subsequently, the supernatants were collected, and LDH release was quantified at 490 nm using the LDH Assay Kit (Beyotime, China).

### Human whole-blood killing assay

The Newman strain was cultured to logarithmic phase in TSB without or with isorhapontigenin (1, 25, and 50 µM) and resuspended in sterile PBS to a concentration of 1.5 × 10^8^ CFU/mL. The bacterial suspension was gently mixed with fresh whole blood collected from healthy volunteers at a ratio of 1:9 in 1.5 mL Eppendorf tubes (500 µL). The tubes were incubated at 37°C for 3 h. Bacterial counts were determined by serial dilution and plating on tryptic soy agar. The experiment was performed in triplicate.

### Intra-macrophage survival assay

RAW264.7 murine macrophage-like cells were cultured in DMEM supplemented with 10% fetal bovine serum at 37°C and 5% CO_2_. Approximately 1 × 10^5^ cells were seeded into 12-well plates and washed twice with PBS before use. The Newman strain was cultured to logarithmic phase in TSB without or with isorhapontigenin (1, 25, and 50 µM) and resuspended in serum-free DMEM. The cells were then infected with the bacteria at a multiplicity of infection of 10:1 and incubated at 37°C for 1 h. After 1 h, the cells were washed three times with PBS, and DMEM containing 10 µg/mL lysostaphin (Sigma) and 100 µg/mL gentamicin (Sigma) was added to each well to kill extracellular bacteria. The culture plates were incubated for an additional hour. After incubation, the cells were washed with PBS and further incubated in fresh DMEM. At 2, 4, and 6 h post-infection, the infected cells were washed three times with PBS to remove extracellular bacteria and dead cells, and 0.05% Triton X-100 (Sigma) was added to lyse the cells. The number of intracellular bacteria was determined by serial dilution and plating on tryptic soy agar.

### Real-time fluorescence quantitative PCR (RT-qPCR)

The *S. aureus* Newman was cultured in TSB at 37°C for 24 h with isorhapontigenin at concentrations of 0 (control), 25, and 50 µM. Cells were pelleted by centrifugation at 8,000 rpm for 2 minutes, and the supernatants were removed. Total RNA was extracted from the bacterial pellets using the Total RNA Purification Kit (Sangon Biotech), according to the manufacturer’s instructions. Reverse transcription was carried out using the PrimeScript RT Kit (Takara). Quantitative PCR was performed with the SYBR Green Pro Taq HS Premix (Ecory Bioengineering Co., Ltd., Hunan, China) on the QuantStudio 5 Real-Time PCR system. Gene expression was normalized to the reference gene *gyrB*, and relative RNA expression changes were calculated using the 2^-ΔΔCt^ method (24). The sequences of the primers used are provided in Table 1. All reactions were performed in triplicate.

**TABLE 1:** Primer pairs used in this study

Primer	Sequence (5´–3´)
*gyrB*-RT-F	ACATTACAGCAGCGTATTAG
*gyrB*-RT-R	CTCATAGTGATAGGAGTCTTCT
*hla*-RT-F	TATTAGAACGAAAGGTACCA
*hla*-RT-R	ACTGTACCTTAAAGGCTGAA
*saeR*-RT-F	GTCGTAACCATTAACTTCTG
*saeR*-RT-R	ATCGTGGATGATGAACAA
*coa*-RT-F	AAGAATACAATGAACTACAG
*coa*-RT-R	CACCATAATATGATACAACT
*mgrA*-RT-F	AACGAATGGAACAAGTAG
*mgrA*-RT-R	ACCTAATAAGCGATTAAGTT
*spa*-RT-F	GAAGATGGTAACGGAGTA
*spa*-RT-R	TGCTTCTTATCAACAACAA
*agrA*-RT-F	GCAGTAATTCAGTGTATGTTCA
*agrA*-RT-R	TATGGCGATTGACGACAA
*RNAIII*-RT-F	TTCACTGTGTCGATAATCCA
*RNAIII*-RT-R	TGATTTCAATGGCACAAGAT
MgrA-exp-F	GCAGCCATATGTCTGATCAACATAATTTAAAAGAAC
MgrA-exp-R	GTAGGCACTCGAGTTATTTTTCCTTTGTTTCATC
*hla*-EMSA-F	CGGAATTAAATCAATTAATTAACTATTAAATAAAAATTAACTATATATGC
*hla*-EMSA-R	GCATATATAGTTAATTTTTATTTAATAGTTAATTAATTGATTTAATTCCG

### Expression and purification of MgrA protein

The full-length *mgrA* gene was PCR-amplified from the genome of *S. aureus* Newman using specific primers. The primers were provided in Table 1, containing NdeI and XhoI restriction sites for cloning purposes, respectively. The amplified PCR product and the pET28a vector were separately digested with the NdeI and XhoI enzymes. The digested fragments were then ligated using T4 DNA ligase to construct the recombinant plasmid. This plasmid was initially transformed into *Escherichia coli* DH5α cells for amplification and sequence verification. Following confirmation, it was subsequently transformed into *E. coli* BL21 (DE3) cells for protein production. Cultures of *E. coli* BL21 (DE3) harboring the plasmid were grown until an OD_600_ of 0.6–0.8 was reached. Protein expression was induced by adding 0.5 mM isopropyl β-D-1-thiogalactopyranoside followed by overnight incubation at 16°C. Post-induction, cells were pelleted via centrifugation and resuspended in lysis buffer containing 20 mM Tris-HCl (pH 8.0), 1 mM dithiothreitol, and 0.5 M NaCl, and supplemented with 1 mM phenylmethylsulfonyl fluoride just before use. The cell suspension was lysed using a high-pressure cell disruptor. The lysate was cleared by centrifugation, and the supernatant was applied to a Ni-NTA affinity column pre-equilibrated with lysis buffer. The column was washed in a stepwise manner with lysis buffer containing increasing concentrations of imidazole (10, 20, and 40 mM). Finally, MgrA protein was eluted with lysis buffer containing a higher concentration of imidazole (300 mM). The eluted fractions were pooled and dialyzed overnight against dialysis buffer (20 mM Tris-HCl [pH 8.0], 1 mM DTT, 50 mM NaCl) to remove excess imidazole. The dialyzed protein was concentrated using an Amicon Ultra centrifugal filter unit and stored at −80°C for future use.

### Electrophoretic mobility shift assay (EMSA)

For the DNA-binding assay, a 50 bp biotin-labeled promoter fragment of the *hla* gene (sequences provided in Table 1) was utilized. MgrA protein at a concentration of 5 µM was incubated with 0.2 µM of the DNA fragment and varying concentrations of isorhapontigenin at 0, 1, 25, and 50 µM. The binding reactions were performed in a 20 µL volume of binding buffer at room temperature for 20 minutes. Prior to sample loading, the 6.5% polyacrylamide gel was pre-electrophoresed at 100 V in 0.5× Tris-borate-EDTA (TBE) buffer at 4°C for 30 minutes. After incubation, the samples were loaded onto the gel and electrophoresed at 100 V for approximately 50 minutes. Post-electrophoresis, the gels were visualized by staining with StarStain Red Nucleic Acid Dye stain.

### Thermal shift assay (TSA)

The TSA was conducted by initially diluting SYPRO Protein Gel Stain (Sigma) in TSA buffer (10 mM Tris, 150 mM NaCl, pH 7.5) to a final staining concentration of 5×. MgrA protein and isorhapontigenin were added to a total reaction volume of 20 µL, with the final concentration of MgrA protein at 5 µM and the final concentration of isorhapontigenin at 50 µM. The mixture was gently mixed and sealed with aluminum foil. The assay conditions were set to a 25°C pre-treatment for 3 minutes, followed by a ramp from 25°C to 95°C at 1°C/min. Fluorescence readings were recorded every 10 seconds for each well (excitation: 490 nm, emission: 520 nm). A control group without drug addition was included, and the experiment was performed in triplicate.

### Molecular docking

The structure of target protein MgrA (ID: 2BV6) was taken from the Research Collaboratory for Structural Bioinformatics Protein Data Bank (RSCB PDB database, http://www.rcsb.org/). Then, it was imported into PyMol 2.3.0 software to add hydrogen and remove both small molecules and water molecules. The structure of isorhapontigenin (PubChem CID: 5318650) was downloaded from the PubChem database. The default root, rotatable bonds, and torsions of the ligand were set in AutoDock Tools 1.5.7 (25). Docking pocket was performed with AutoDock 4 using a grid box of 40 × 40 × 40 dimensions and centered at *x* = 80.0, *y* = 4.9, and *z* = 4.5. The structure visualization used for molecular docking was done with PyMol 2.3.0 (26).

### Mice skin abscess model

Six-week-old female BALB/c mice weighing 18–20 g were divided into three groups with five mice each. The *S. aureus* Newman was cultured to logarithmic phase, harvested, washed with PBS, and resuspended to 1.5 × 10^8^ CFU/mL. Mice were subcutaneously injected with 100 µL of the Newman suspension (1.5 × 10^7^ CFU). At 12 h post-infection, the treatment group began receiving intraperitoneal injections of isorhapontigenin (100 µL at 20 mg/kg) every 24 h for 3 days, while the control group received PBS. The size of the abscesses was measured daily using calipers, with length (L) and width (W) recorded to calculate the area (A) as A = π (L × W)/2. All animals were euthanized 5 days post-infection.

### MRSA-infected pneumonia murine model

Six-week-old female BALB/c mice weighing 18–20 g were similarly divided into three groups of 10 mice each. The *S. aureus* Newman was cultured overnight in TSB, and then inoculated into fresh TSB to reach the logarithmic growth phase. Bacterial cells were harvested, washed, and resuspended in sterile PBS to a final concentration of 1.5 × 10^10^ CFU/mL. Mice were anesthetized with pentobarbital sodium and intranasally inoculated with 20 µL of the bacterial suspension. Two hours later, mice received intraperitoneal injections of isorhapontigenin (100 µL at 20 mg/kg) or PBS every 24 h for 3 days. Morbidity was monitored over 3 days, after which mice were euthanized, and right lungs were collected for bacterial burden analysis. The right lung tissues were homogenized, serially diluted, and plated on tryptic soy agar for colony counting after overnight incubation. The left lungs were fixed in formalin, and following standard histological procedures, which included paraffin embedding and sectioning, the tissues were stained with hematoxylin and eosin (H&E). Stained sections were examined under a light microscope to assess histopathological changes.

### Statistical analysis

All statistical analyses were performed using GraphPad Prism 8. Unpaired two-tailed Student’s *t*-test and χ^2^ test were employed for comparisons between two groups, where appropriate. *P*-value <0.05 was considered statistically significant. Data are presented as mean ± standard deviation. The following notations indicate statistical significance levels: *P < 0.05, **P < 0.01, ***P < 0.001, ****P < 0.0001.

## RESULTS

### Isorhapontigenin demonstrates favorable biocompatibility

**Fig 1:**
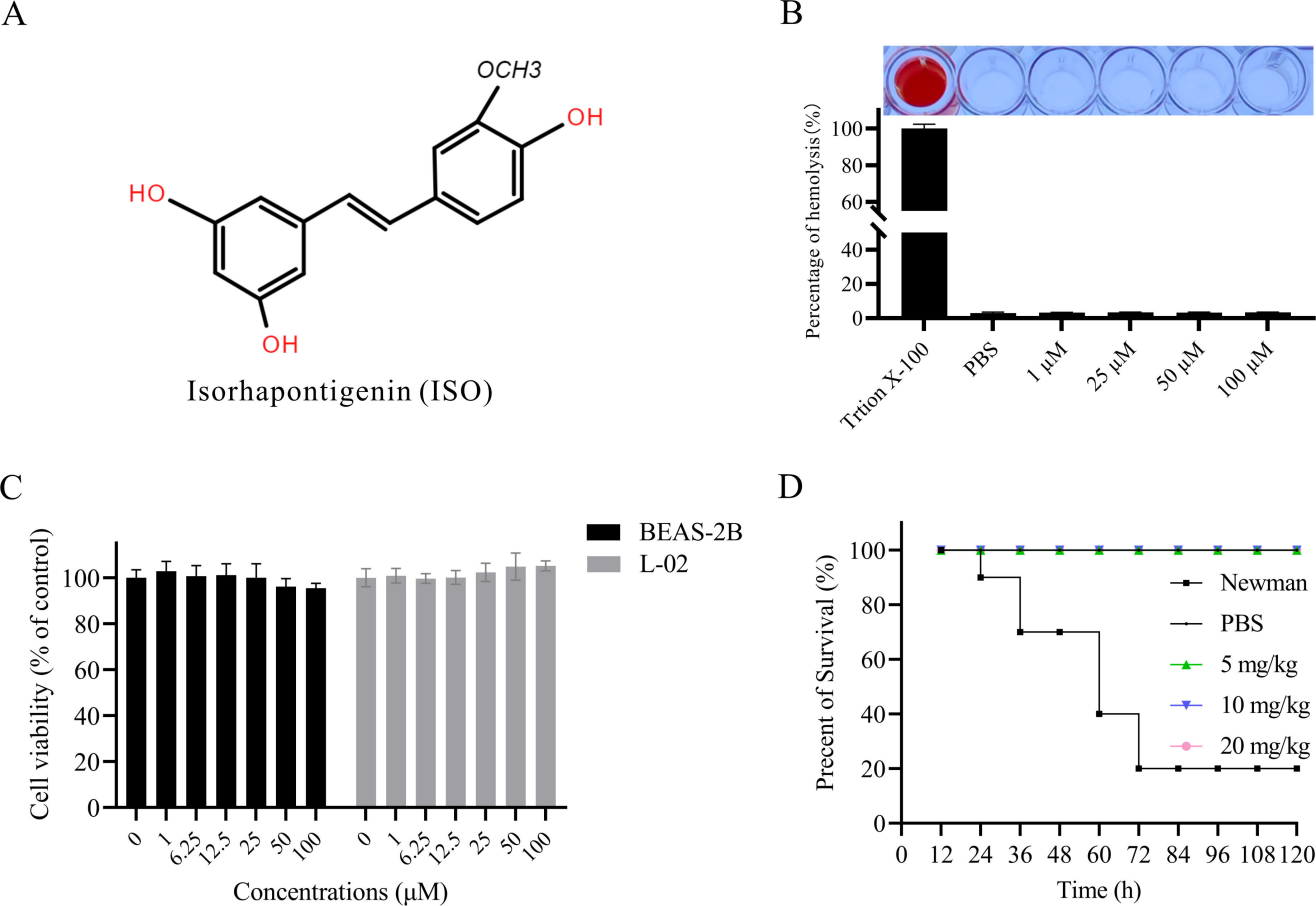
*In vitro* and *in vivo* toxicity of isorhapontigenin (ISO). (**A**) The chemical structures of isorhapontigenin. (**B**) Hemolytic activity of isorhapontigenin on rabbit erythrocytes. Positive control: 2% Triton X-100; negative control: PBS. (**C**) Cell viability of BEAS-2B and L-02 cell lines after 24-h exposure to various concentrations of isorhapontigenin, relative to untreated control cells (set at 100% viability). (**D**) Survival rates of *G. mellonella* larvae after administration of isorhapontigenin, with 10 individuals per treatment group. The positive control group received injections of *S. aureus* Newman, and the negative control group received PBS injections.

Isorhapontigenin, a stilbenoid compound structurally akin to resveratrol, is differentiated by its methoxylation (Fig. 1A). To ascertain the biosafety of isorhapontigenin, we performed hemolysis, cell cytotoxicity, and *G. mellonella* larvae toxicity assays. Incubation of varying concentrations of isorhapontigenin with rabbit red blood cells did not result in significant hemoglobin liberation in the supernatant when compared with the PBS control (Fig. 1B), suggesting that isorhapontigenin does not induce hemolysis at the tested concentrations. The cytotoxic effects of isorhapontigenin on BEAS-2B and L-02 cells were evaluated following 24-h exposure utilizing the MTT assay. Figure 1C illustrates that concentrations of isorhapontigenin up to 100 µM did not manifest discernible cytotoxic effects on BEAS-2B and L-02 cells. Additionally, isorhapontigenin was administered intraperitoneally at doses of 5, 10, and 20 mg/kg to *G. mellonella* larvae, and survival was monitored over 120 h. Consistent with the *in vitro* findings, isorhapontigenin did not elicit acute toxicity (Fig. 1D). In summary, our experimental data indicate that isorhapontigenin demonstrates favorable biocompatibility and holds considerable promise for therapeutic applications.

### Isorhapontigenin effectively inhibits hemolytic ability of *S*. *aureus*

**Fig 2:**
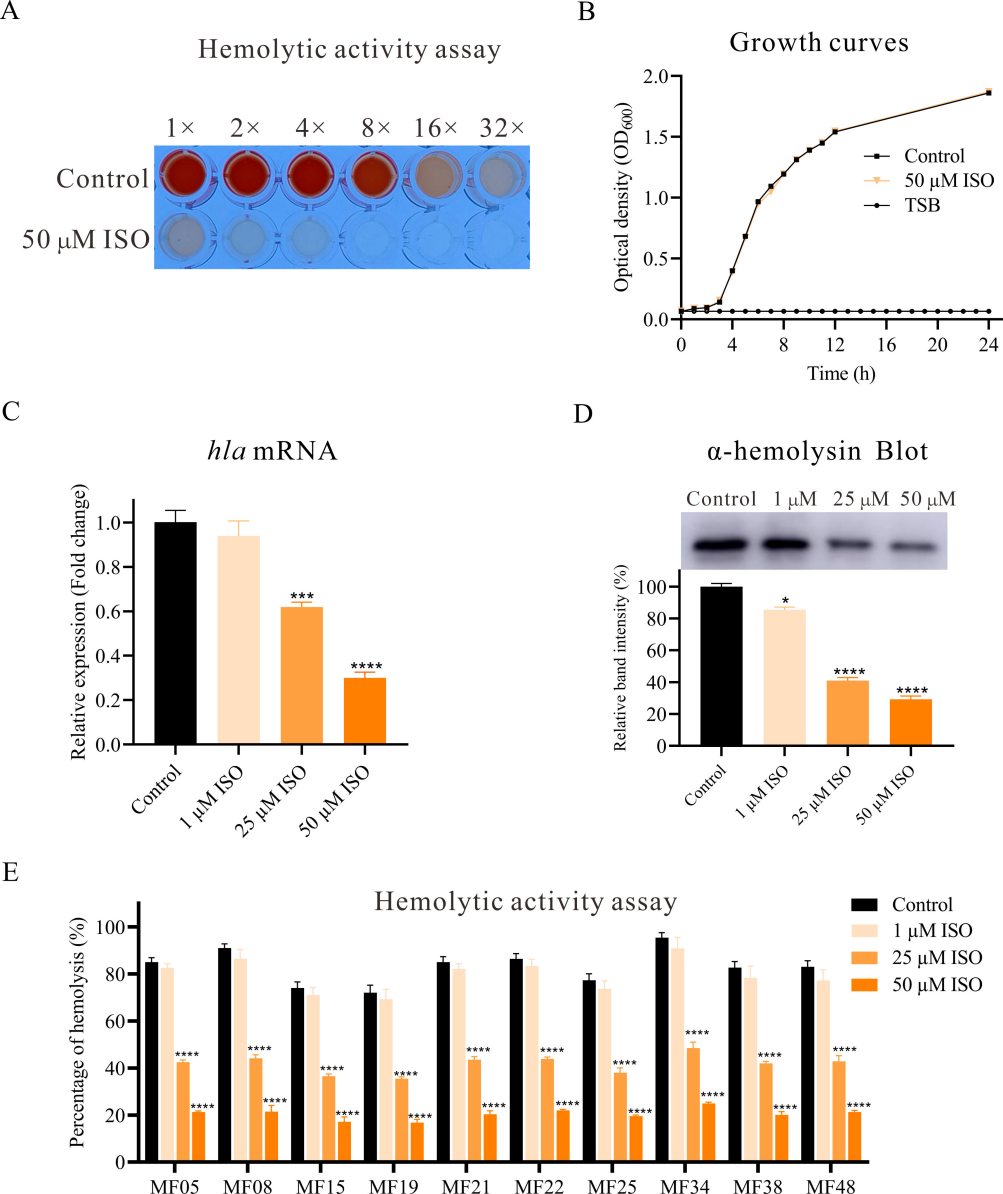
Effect of isorhapontigenin (ISO) on the hemolytic activity of *S. aureus*. (**A**) The effects of isorhapontigenin on the hemolytic activity of *S. aureus* Newman. Dilute the supernatant from bacterial cultures, both isorhapontigenin-treated and untreated, by a specified factor ×-fold dilution, and incubate with a 5% RBC suspension. Positive control: 2% Triton X-100; negative control: PBS. (**B**) Growth curves of *S. aureus* Newman cultured in the presence of 50 µM isorhapontigenin. DMSO serves as a control to exclude the effects of the solvent on bacterial growth, with TSB as a negative control. (**C**) RT-qPCR analysis of the effect of isorhapontigenin on the expression of the *hla* gene. (**D**) Western blot analysis of the impact of isorhapontigenin on the production of α-hemolysin. (**E**) Evaluation of isorhapontigenin’s effect on the hemolytic activity of clinical *S. aureus* isolates. Positive control: 2% Triton X-100; negative control: PBS.

To confirm whether isorhapontigenin, a methoxylated analog of resveratrol, possesses similar anti-virulence functionality, we conducted hemolysis assays. The release of hemoglobin from erythrocytes in response to hemolysin was quantified spectrophotometrically. RBCs were incubated with serial dilutions of *S. aureus* culture supernatants, and absorbance was recorded following incubation. As shown in Fig. 2A, the control group’s supernatant caused complete hemolysis even at an eightfold dilution. In contrast, treatment with 50 µM isorhapontigenin resulted in a significant decrease in hemolytic activity, even with undiluted supernatants. The MIC of isorhapontigenin against *S. aureus* Newman and 10 clinical isolates exceeded 500 µM, as determined by the microbroth dilution method. Growth curve results revealed that isorhapontigenin at a concentration of 50 µM did not notably impact the growth of *S. aureus* Newman (Fig. 2B). Therefore, we utilized concentrations of isorhapontigenin up to 50 µM in subsequent experiments to ensure the observed effects on *S. aureus* biological functions were not due to growth inhibition.

RT-qPCR results confirmed that isorhapontigenin significantly reduces the transcription levels of the *hla* gene encoding α-hemolysin in a dose-dependent manner (Fig. 2C). To further elucidate the effects of isorhapontigenin on α-hemolysin expression, *S. aureus* Newman was treated with 1 µM, 25 µM, and 50 µM of isorhapontigenin. Post-treatment, the intensity of bands corresponding to α-hemolysin on gels demonstrated a marked reduction compared to the untreated control (Fig. 2D). Grayscale analysis confirmed that treatment with 50 µM isorhapontigenin significantly diminished α-hemolysin expression (*P* < 0.001), supporting the potent inhibitory effect of 50 µM isorhapontigenin on the hemolytic activity of *S. aureus* Newman. To validate the generalizability of isorhapontigenin anti-hemolytic activity, we examined its effect on the hemolytic activities of five clinical MSSA and five MRSA isolates. Isorhapontigenin consistently inhibited the hemolytic activities of both MSSA and MRSA isolates in a dose-dependent manner (Fig. 2E), which reinforces its potential as a broad-spectrum inhibitor of *S. aureus* virulence.

### Isorhapontigenin reduces *S. aureus* cytotoxicity and impairs its immune evasion capability

**Fig 3:**
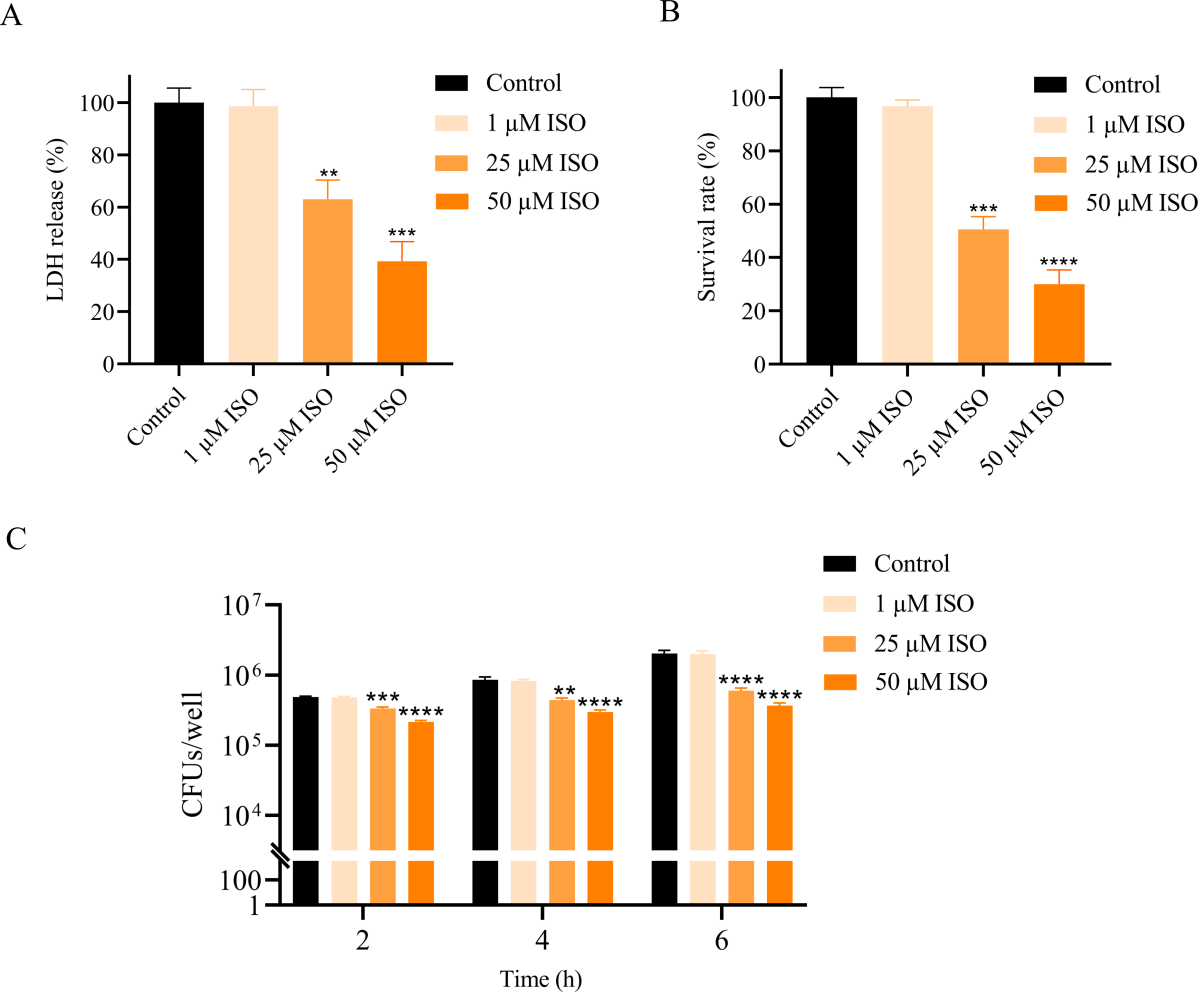
Effect of isorhapontigenin (ISO) on the cytotoxicity and immune evasion capabilities of *S. aureus*. (**A**) The effects of isorhapontigenin on LDH release from BEAS-2B cells after infection with *S. aureus* for 24 h. LDH release is expressed as a percentage of the maximal release induced by untreated *S. aureus* Newman. (**B**) Effect of isorhapontigenin on *S. aureus* survival in whole blood. Survival rates are presented as percentages relative to the maximal survival of untreated *S. aureus* Newman. (**C**) Treatment with isorhapontigenin decreases *S. aureus* survival in RAW264.7 cells.

*S. aureus* is a significant respiratory pathogen. We assessed the protective role of isorhapontigenin against *S. aureus* cytotoxicity toward bronchial epithelial BEAS-2B cells using an LDH release assay. The assay demonstrated a dose-dependent decrease in LDH release upon treatment with varying concentrations of isorhapontigenin in the presence of *S. aureus* Newman (Fig. 3A). Specifically, LDH release was mitigated to 63.06% of the control level at 25 µM of isorhapontigenin (*P* < 0.01), and further reduced to 39.27% at 50 µM (*P* < 0.001), indicating improved cell viability. *S. aureus* employs several strategies to evade host defenses, including the secretion of α-hemolysin and immune system evasion. We explore the capability of isorhapontigenin to impair *S. aureus* survival post-exposure to human whole blood. The findings indicated that 50 µM of isorhapontigenin significantly decreased the survival rate of *S. aureus* Newman to 30.05% compared to the untreated control (Fig. 3B).

Macrophages play an essential role in innate immunity by facilitating phagocytosis and the elimination of pathogens. We assessed the impact of isorhapontigenin on *S. aureus* survival within macrophages. Notably, the survival rate of *S. aureus* within RAW264.7 macrophages was substantially reduced after exposure to 50 µM isorhapontigenin over 2-h, 4-h, and 6-h periods (Fig. 3C). The treatment resulted in a significant decline in the 6-h survival of *S. aureus* Newman from 2.01 × 10^6^ to 3.7 × 10^5^ CFU. These findings suggest that isorhapontigenin enhances the immune-mediated clearance of *S. aureus* and reduces its resistance to macrophage phagocytosis.

### Isorhapontigenin attenuating *S. aureus* virulence through MgrA-mediated regulation

**Fig 4:**
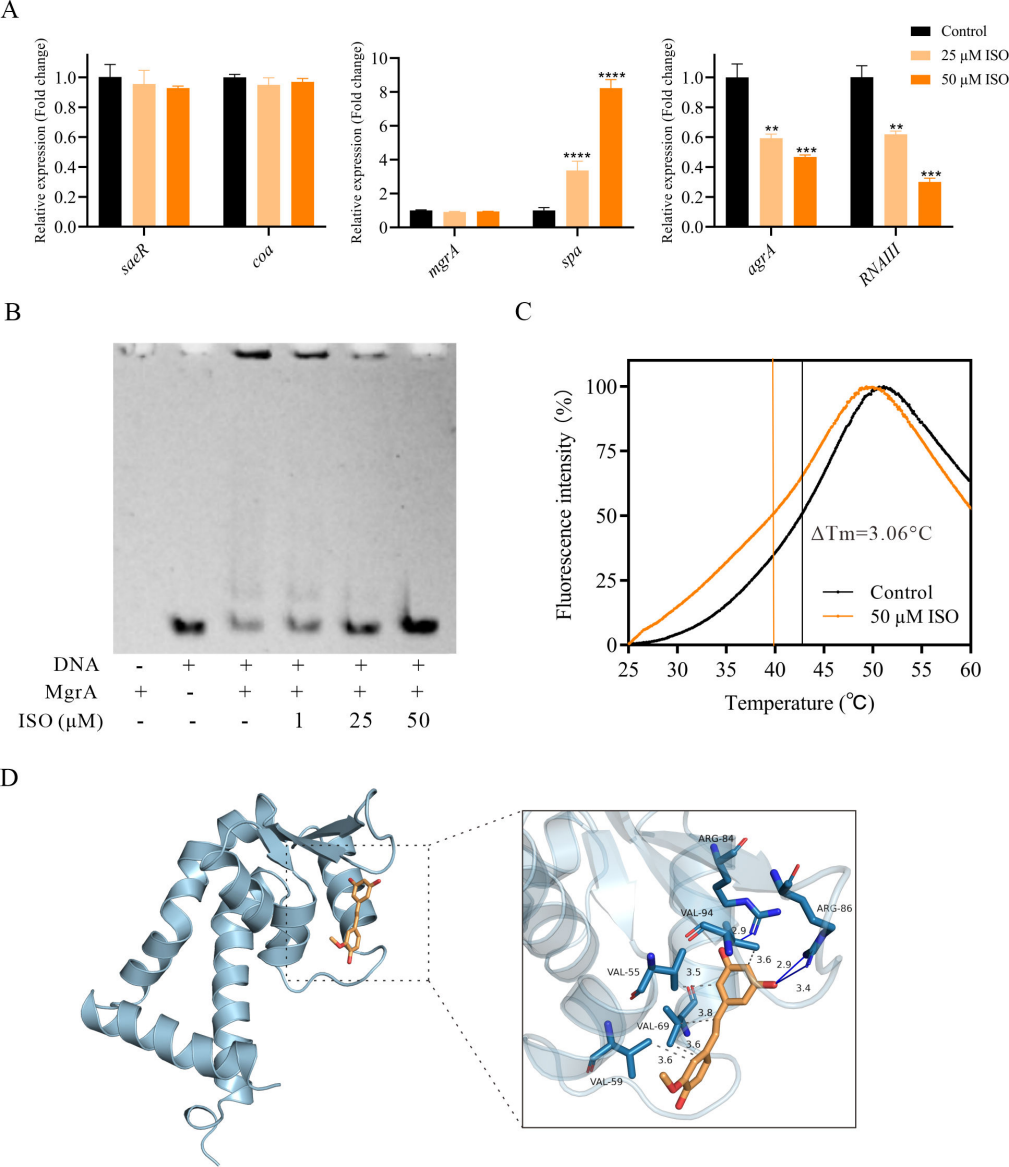
Isorhapontigenin (ISO) attenuating *S. aureus* virulence through MgrA-mediated regulation. (**A**) RT-qPCR analysis of the effect of isorhapontigenin on key virulence regulators and their target genes. (**B**) EMSA analysis of the impact of isorhapontigenin on MgrA protein binding to *hla* promoter. Lane 1 shows EMSA with protein only; lane 2 shows EMSA with *hla* DNA only; lanes 3–6 show EMSA with increasing concentrations of isorhapontigenin (0, 1, 25, and 50 µM). (**C**) TSA analysis of the interaction between isorhapontigenin and MgrA protein. (**D**) Molecular docking analysis of MgrA protein with isorhapontigenin.

The virulence of *S. aureus*, particularly its production of α-hemolysin, is predominantly controlled by transcriptional regulators including SaeR, MgrA, and the Agr quorum-sensing system. To elucidate the mechanism underlying isorhapontigenin’s attenuation of α-hemolysin activity, we investigated the effect of isorhapontigenin on key genes responsible for *hla* expression regulation: *saeR* and its target gene *coa*, *mgrA* and its target gene *spa*, as well as *agrA* and its target gene *RNAIII*. Although both SaeR and AgrA can influence the *coa* transcript, and *spa* is also regulated by Agr system, our RT-qPCR data show distinct patterns of gene expression modulation by isorhapontigenin (Fig. 4A). Notably, while *mgrA* transcription was unaffected, its target gene *spa* showed a significant concentration-dependent upregulation, suggesting that isorhapontigenin may directly interact with MgrA. Conversely, both *agrA* and *RNAIII* transcription levels were significantly suppressed, indicating that isorhapontigenin’s impact on α-hemolysin production may involve multiple regulatory pathways, including potential modulation of the Agr system through an MgrA-mediated pathway. To further investigate the impact of isorhapontigenin on MgrA’s functionality, we performed gel mobility shift assays with his-tagged MgrA protein in the presence of isorhapontigenin. Figure 4B shows that isorhapontigenin at concentrations of 25 µM and 50 µM substantially reduced MgrA’s binding affinity to the *hla* promoter, which supports the hypothesis of a direct regulatory effect exerted by isorhapontigenin.

Thermal shift assay (TSA) was conducted to examine potential interactions between isorhapontigenin and the MgrA protein, as indicated by shifts in the protein’s melting temperature (Tm). A change in Tm (ΔTm) greater than 2°C generally signifies significant binding. TSA results revealed that the Tm of MgrA decreased from 42.68°C to 39.62°C in the presence of 50 µM isorhapontigenin (Fig. 4C). This suggests a direct interaction between isorhapontigenin and MgrA protein, thereby leading to the inhibition of MgrA activity. This finding aligns with the results of RT-qPCR and sheds light on the stable levels of *mgrA* mRNA. It suggests that isorhapontigenin’s interaction with the MgrA protein directly influences its function, rather than affecting the expression of the *mgrA* mRNA.

Additionally, molecular docking studies indicate that isorhapontigenin binds to the MgrA protein of *S. aureus* via hydrophobic forces and hydrogen bonding. Notably, isorhapontigenin interacts with active sites of MgrA with a binding energy of −6.50 kcal/mol and exhibited two hydrogen bonds (ARG-84 and ARG-86) and four hydrophobic force (VAL-55, VAL-59, VAL-69, and VAL-94), indicating a strong interaction between isorhapontigenin and MgrA (Fig. 4D). The strong binding efficacy of isorhapontigenin with MgrA confirmed its potential as an anti-virulence agent.

### Isorhapontigenin significantly decreased the pathogenicity of *S. aureus*

**Fig 5:**
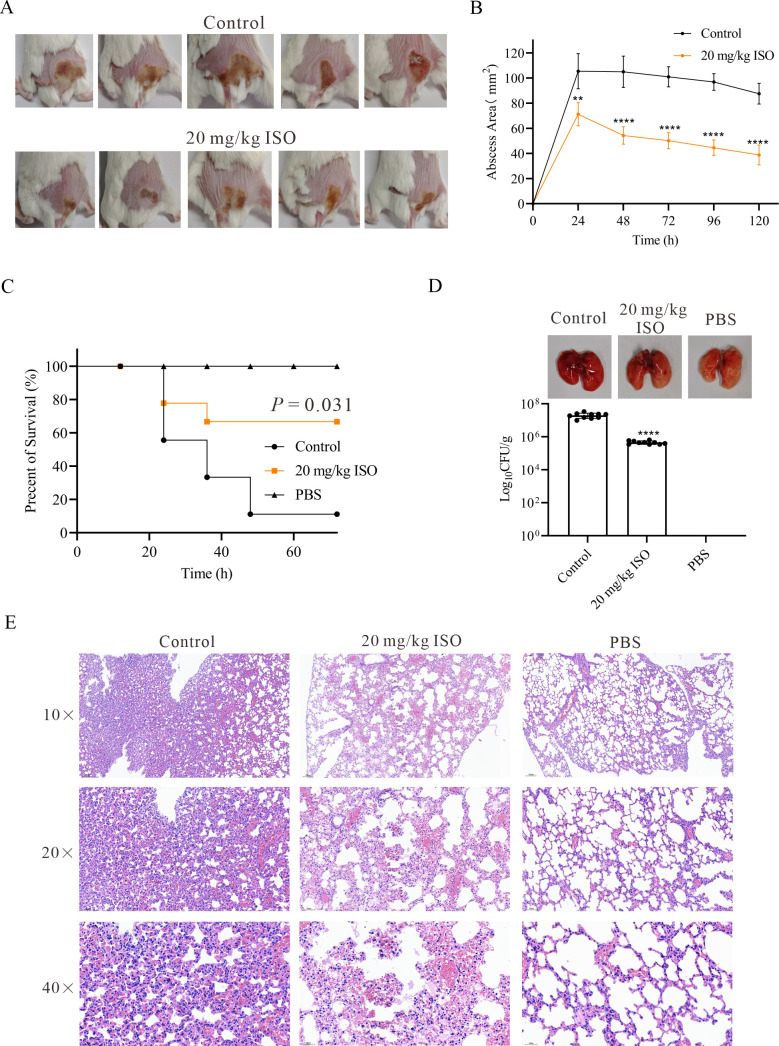
Therapeutic effects of isorhapontigenin (ISO) against *S. aureus* Newman-induced skin abscess and pneumonia in mice. (**A**) Skin lesions resulting from *S. aureus* infection (photographed on day 3 post-infection). (**B**) Comparison of abscess size (area) between Newman (control) and isorhapontigenin-treated groups (treated with 20 mg/kg per day). (**C**) The impact of isorhapontigenin on survival rates of mice (*n* = 10) infected with a lethal dose of *S. aureus* Newman. (**D**) Gross evaluation of lungs in all mice and determination of bacterial load in lung tissue. (**E**) Histological analysis of the impact of isorhapontigenin on the lungs of mice, using H&E staining.

In the mouse skin abscess model, the administration of bacterial suspensions subcutaneously into the dorsa led to abscess formation. Notably, abscesses in the group treated with isorhapontigenin were significantly smaller compared to the control group (Fig. 5A). The changes in the size of Newman strain abscesses in isorhapontigenin-treated groups indicate a reduction in abscess size over time following isorhapontigenin administration (Fig. 5B).

Moreover, we developed a pneumonia model to investigate the protective effects of isorhapontigenin against *S. aureus*-induced pneumonia in mice. Mice were inoculated intranasally with 2 × 10^8^ CFU of *S. aureus* Newman strain and were monitored every 12 h, and mortality was recorded. The control group exhibited a cessation in mortality after 48 h post-infection, yet the mortality rate reached 90% at that juncture. In contrast, the group treated with isorhapontigenin demonstrated a mortality rate of only 30% at the same time point. Statistical analysis revealed that at 72 h post-infection, the mortality rate in the control group remained at 90%, while subcutaneous injection of 20 mg/kg isorhapontigenin significantly reduced the mortality rate to 30%, thereby improving survival by 60% (*P* = 0.031, Fig. 5C). Additionally, post-dissection of the mice, we quantified the bacterial load in their lungs. The bacterial load in the isorhapontigenin-treated group was markedly lower than in the control group (4.58 × 10^5^ CFU/g vs 2.01 × 10^7^ CFU/g) (Fig. 5D). Upon examination of the overall changes, the lungs of the control group mice exhibited severe congestion, edema, and widespread hemorrhagic lesions. Conversely, isorhapontigenin treatment resulted in a notable improvement in pulmonary congestion and hemorrhage. Histological analysis confirmed these findings (Fig. 5E). In the control group, alveolar spaces were densely filled with inflammatory cells, indicative of severe inflammation. In contrast, mice treated with isorhapontigenin demonstrated a significantly milder inflammatory response, characterized by a noticeable reduction in the accumulation of inflammatory cells within the alveolar spaces. In conclusion, isorhapontigenin has been proven to be an effective agent in alleviating lung tissue damage induced by *S. aureus*, thereby increasing the survival rate of the mice.

## DISCUSSION

The pivotal role of α-hemolysin in the pathogenesis of *S. aureus* infections is well-documented across numerous studies (27–29). Developing inhibitors that target virulence factors represents an innovative approach to combating bacterial infections, showing particular promise against multidrug-resistant pathogens (30). Traditional antibiotics apply significant selective pressure on bacterial populations during treatment, often accelerating the emergence of resistance. In contrast, the advantage of anti-virulence therapies resides in their specificity for virulence determinants that are non-essential for bacterial proliferation. Consequently, such therapies attenuate bacterial virulence without imposing selective pressures, thereby potentially curtailing the development of resistance. Given the multifactorial nature of *S. aureus* virulence, targeting a single virulence factor may not suffice for effective infection control. Nonetheless, the transcriptional regulator MgrA is an upstream effector that modulates multiple virulence genes. Inhibiting MgrA activity could therefore exert a widespread influence on the pathogenicity of *S. aureus*.

The *G. mellonella* larvae model has been proven to be a highly reliable model for evaluating drug toxicity (31). In this study, we demonstrated the good biocompatibility of isorhapontigenin through hemolysis assays, cytotoxicity assays, and *G. mellonella* larvae toxicity experiments. Previous studies have shown that resveratrol can reduce the virulence of *S. aureus* by downregulating the expression of Hla (11, 12). Considering the structural similarity between isorhapontigenin and resveratrol, we postulated that isorhapontigenin might exhibit analogous anti-virulence capabilities. Hemolysis activity assays confirmed that 50 µM of isorhapontigenin markedly inhibited the hemolytic activity of both the *S. aureus* Newman and various clinical isolates, underscoring the broad applicability of isorhapontigenin’s anti-virulence effects. RT-qPCR and Western blot analyses further revealed that isorhapontigenin significantly inhibits *hla* transcription and α-hemolysin protein expression in a dose-dependent manner. Additionally, we observed that isorhapontigenin diminished *S. aureus* toxicity toward BEAS-2B cells and the bacterium’s resistance to eradication by human whole blood. α-Hemolysin is known to facilitate *S. aureus* intracellular survival by promoting non-canonical autophagy pathways dependent on Ca^2+^ and autophagy-related gene 5 (Atg5) or by mediating escape from phagosomes, both reliant on its pore-forming activity (24, 32). Thus, we quantified the intracellular bacterial count at 2 h, 4 h, and 6 h post-infection in RAW264.7 macrophages. The findings indicate that isorhapontigenin treatment effectively reduced the intracellular survival of *S. aureus*, suggesting an impairment of α-hemolysin’s pore-forming function and its associated survival pathways. This insight bears considerable significance for the clinical management of *S. aureus* infections and posits isorhapontigenin as a potential new anti-virulence drug candidate.

Hla is a critical virulence determinant in the pathogenesis of *S. aureus*, associated with necrotizing pneumonia, skin and soft tissue infections, and sepsis (1). The regulation of the *hla* gene is complex, involving multiple factors rather than a solitary genetic element. The expression of *hla* is controlled by the positive regulators such as AgrA, MgrA, and SaeR (6, 33) and the negative regulators such as Rot and SarT (34–36). MgrA, a key transcriptional regulator within the MarR/SarA protein family, orchestrates the expression of multiple virulence factors in *S. aureus*, including α-hemolysin, the capsule, and proteases (6, 37). The vital role of MgrA in *S. aureus* virulence has been substantiated by animal model studies (38–40). MgrA can not only indirectly promote the expression of the *hla* gene by regulating *RNAIII*, but also directly bind to the *hla* promoter to increase its expression (6). Protein-DNA interactions are fundamental to the mechanics of transcriptional regulation (41). EMSAs are a staple technique for assessing the binding affinity between transcription factors and DNA sequences and can be adapted to identify small molecule inhibitors by co-incubation in the assay (42). To investigate whether isorhapontigenin directly interacts with MgrA, we conducted EMSA and thermal shift assays. The EMSA results indicated that isorhapontigenin could inhibit the binding of MgrA to *hla* promoter. Complementarily, thermal shift assays corroborated the direct interaction between isorhapontigenin and MgrA. Molecular docking was utilized to simulate the potential binding conformations of isorhapontigenin with MgrA, identifying several key amino acid residues possibly involved in this interaction. This series of findings suggests that isorhapontigenin has the potential to serve as an anti-virulence drug for treating *S. aureus* infections by inhibiting the function of MgrA, which plays a crucial role in regulating the virulence of *S. aureus*. Future investigations should incorporate site-directed mutagenesis to validate the specific amino acid residues of MgrA that interact with isorhapontigenin. Nonetheless, the possibility of direct interactions between isorhapontigenin and other regulatory proteins cannot be completely ruled out. Understanding these potential interactions is crucial for fully elucidating isorhapontigenin’s mechanism of action and optimizing its therapeutic efficacy. Further studies are essential to explore these interactions and advance our comprehension of isorhapontigenin’s potential in medical applications.

*S. aureus* is a common etiological agent of purulent skin infections (43). In a mouse skin abscess model, isorhapontigenin administration resulted in a significant diminution of the abscess area caused by *S. aureus*, underscoring its therapeutic potential. Moreover, *S. aureus* pneumonia, characterized by acute purulent pulmonary inflammation, typically presents with sluggish antibiotic efficacy, protracted convalescence, and high morbidity and mortality rates (44). In mouse pneumonia model, isorhapontigenin conferred protective effects against lethal *S. aureus*-induced infection, markedly mitigating pulmonary congestion and inflammation and substantially decreasing the bacterial load in the lungs.

In conclusion, isorhapontigenin exhibits significant efficacy in reducing *S. aureus* virulence by inhibiting the transcriptional regulator MgrA. These findings highlight its potential as an anti-virulence therapeutic agent. However, further comprehensive investigations are necessary to confirm the clinical safety and efficacy of isorhapontigenin. If isorhapontigenin or other analogs targeting MgrA can be successfully developed into pharmaceutical agents, they could serve as potent tools against infections caused by *S. aureus*.
